# Osteoarthritis: toward a comprehensive understanding of pathological mechanism

**DOI:** 10.1038/boneres.2016.44

**Published:** 2017-01-17

**Authors:** Di Chen, Jie Shen, Weiwei Zhao, Tingyu Wang, Lin Han, John L Hamilton, Hee-Jeong Im

**Affiliations:** 1 Department of Biochemistry, Rush University Medical Center, Chicago, IL, USA; 2 Department of Orthopaedic Surgery, Washington University, St Louis, MO, USA; 3 Department of Orthopaedics & Traumatology, Li Ka Shing Faculty of Medicine, The University of Hong Kong, Hong Kong, China; 4 Department of Pharmacy, Shanghai Ninth People’s Hospital, Shanghai JiaoTong University School of Medicine, Shanghai, China; 5 School of Biomedical Engineering, Science, and Health Systems, Drexel University, Philadelphia, PA, USA

## Abstract

Osteoarthritis (OA) is the most common degenerative joint disease and a major cause of pain and disability in adult individuals. The etiology of OA includes joint injury, obesity, aging, and heredity. However, the detailed molecular mechanisms of OA initiation and progression remain poorly understood and, currently, there are no interventions available to restore degraded cartilage or decelerate disease progression. The diathrodial joint is a complicated organ and its function is to bear weight, perform physical activity and exhibit a joint-specific range of motion during movement. During OA development, the entire joint organ is affected, including articular cartilage, subchondral bone, synovial tissue and meniscus. A full understanding of the pathological mechanism of OA development relies on the discovery of the interplaying mechanisms among different OA symptoms, including articular cartilage degradation, osteophyte formation, subchondral sclerosis and synovial hyperplasia, and the signaling pathway(s) controlling these pathological processes.

## Introduction

Osteoarthritis (OA) is the most common degenerative joint disease, affecting more than 25% of the population over 18 years-old. Pathological changes seen in OA joints include progressive loss and destruction of articular cartilage, thickening of the subchondral bone, formation of osteophytes, variable degrees of inflammation of the synovium, degeneration of ligaments and menisci of the knee and hypertrophy of the joint capsule.^1^ The etiology of OA is multi-factorial and includes joint injury, obesity, aging, and heredity.^1–5^ Because the molecular mechanisms involved in OA initiation and progression remain poorly understood, there are no current interventions to restore degraded cartilage or decelerate disease progression. Studies using genetic mouse models suggest that growth factors, including transforming growth factor-β (TGF-β), Wnt3a and Indian hedgehog, and signaling molecules, such as Smad3, β-catenin and HIF-2α,^6–10^ are involved in OA development. One feature common to several OA animal models is the upregulation of Runx2.^7,8,11–13^ Runx2 is a key transcription factor directly regulating the transcription of genes encoding matrix degradation enzymes in articular chondrocytes.^14–17^ In this review article, we will discuss the etiology of OA, the available mouse models for OA research and current techniques used in OA studies. In addition, we will also summarize the recent progress on elucidating the molecular mechanisms of OA pain. Our goal is to provide readers a comprehensive coverage on OA research approaches and the most up-to-date progress on understanding the molecular mechanism of OA development.

## Etiology

OA is the most prevalent joint disease associated with pain and disability. It has been forecast that 25% of the adult population, or more than 50 million people in the US, will be affected by this disease by the year 2020 and that OA will be a major cause of morbidity and physical limitation among individuals over the age of 40.^18,19^ Major clinical symptoms include chronic pain, joint instability, stiffness and radiographic joint space narrowing.^20^ Although OA primarily affects the elderly, sports-related traumatic injuries at all ages can lead to post-traumatic OA. Currently, apart from pain management and end stage surgical intervention, there are no effective therapeutic treatments for OA. Thus, there is an unmet clinical need for studies of the etiology and alternative treatments for OA. In recent years, studies using the surgically induced destabilization of the medial meniscus (DMM) model and tissue or cells from human patients demonstrated that genetic, mechanical, and environmental factors are associated with the development of OA. At the cellular and molecular level, OA is characterized by the alteration of the healthy homeostatic state toward a catabolic state.

### Aging

One of the most common risk factors for OA is age. A majority of people over the age of 65 were diagnosed with radiographic changes in one or more joints.^21–25^ In addition to cartilage, aging affects other joint tissues, including synovium, subchondral bone and muscle, which is thought to contribute to changes in joint loading. Studies using articular chondrocytes and other cells suggest that aging cells show elevated oxidative stress that promotes cell senescence and alters mitochondrial function.^26–29^ In a rare form of OA, Kashin-Back disease, disease progression was associated with mitochondrial dysfunction and cell death.^30^ Another hallmark of aging chondrocytes is reduced repair response, partially due to alteration of the receptor expression pattern. In chondrocytes from aged and OA cartilage, the ratio of TGF-β receptor ALK1 to ALK5 was increased, leading to down-regulation of the TGF-β pathway and shift from matrix synthesis activity to catabolic matrix metalloproteinase (MMP) expression.^31,32^ Recent studies also indicate that methylation of the entire genomic DNA displayed a different signature pattern in aging cells.^33,34^ Genome-wide sequencing of OA patients also confirmed that this epigenetic alteration occurred in OA chondrocytes,^35–37^ partially due to changes in expression of Dnmts (methylation) and Tets (de-methylation) enzymes.^38–40^

### Obesity

In recent years, obesity has become a worldwide epidemic characterized by an increased body composition of adipose tissue. The association between obesity and OA has long been recognized.^41,42^ Patients with obesity develop OA earlier and have more severe symptoms, higher risk for infection and more technical difficulties for total joint replacement surgery. In addition to increased biomechanical loading on the knee joint, obesity is thought to contribute to low-grade systemic inflammation through secretion of adipose tissue-derived cytokines, called adipokines.^43–45^ Specifically, levels of pro-inflammatory cytokines, including interleukin (IL)-1β, IL-6, IL-8, and tumor necrosis factor alpha (TNF-α) were elevated^46–50^ in high-fat diet-induced mouse obesity models^51–54^ and in obese patients.^55–57^ These inflammatory factors may trigger the nuclear factor-κB (NF-κB) signaling pathway to stimulate an articular chondrocyte catabolic process and lead to extracellular matrix (ECM) degradation through the upregulation of MMPs.^58–60^

### Sport injury

Knee injury is the major cause of OA in young adults, increasing the risk for OA more than four times. Recent clinical reports showed that 41%–51% of participants with previous knee injuries have radiographic signs of knee OA in later years.^61^ Cartilage tissue tear, joint dislocation and ligament strains and tears are the most common injuries seen clinically that may lead to OA. Trauma-related sport injuries can cause bone, cartilage, ligament, and meniscus damage, all of which can negatively affect joint stabilization.^62–66^ Signs of inflammation observed in both patients with traumatic knee OA and in mouse injury models include increased cytokine and chemokine production, synovial tissue expansion, inflammatory cell infiltration, and NF-κB pathway activation.^67^

### Inflammation

It has been established that the chronic low-grade inflammation found in OA contributes to disease development and progression. During OA progression, the entire synovial joint, including cartilage, subchondral bone, and synovium, are involved in the inflammation process.^68^ In aging and diabetic patients, conventional inflammatory factors, such as IL-1β and TNF-α, as well as chemokines, were reported to contribute to the systemic inflammation that leads to activation of NF-κB signaling in both synovial cells and chondrocytes. Innate inflammatory signals were also involved in OA pathogenesis, including damage associated molecular patterns (DAMPs), alarmins (S100A8 and S100A9) and complement.^69–71^ DAMPs and alarmins were reported to be abundant in OA joints, signaling through either toll-like receptors (TLR) or the canonical NF-κB pathway to modulate the expression of MMPs and a disintegrin and metalloprotease with thrombospondin motif (ADAMTS) in chondrocytes.^72–76^ Complement can be activated in OA chondrocytes and synovial cells by DAMPs, ECM fragments and dead-cell debris.^77,78^ Recent studies further clarified that systemic inflammation can re-program chondrocytes through inflammatory mediators toward hypertrophic differentiation and catabolic responses through the NF-κB pathway,^9,10,79^ the ZIP8/Zn^+^/MTF1 axis,^80^ and autophagy mechanisms.^81–85^ Indeed, the recent Kyoto Encyclopedia of Genes and Genomes (KEGG) pathway analyses of OA and control samples provide evidence that inflammation signals contribute to OA pathogenesis through cytokine-induced mitogen-activated protein (MAP) kinases, NF-κB activation, and oxidative phosphorylation.^86^

### Genetic predisposition

An inherited predisposition to OA has been known for many years from family-based studies.^87–89^ Although the genetics of OA are complex, the genetic contribution to OA is highly significant. Over the past decade, the roles of genes and signaling pathways in OA pathogenesis have been demonstrated by *ex vivo* studies using tissues derived from OA patients and *in vivo* studies using surgically induced OA animal models and genetic mouse models. For example, alterations in TGF-β, Wnt/β-catenin, Indian Hedgehog (Ihh), Notch and fibroblast growth factor (FGF) pathways have been shown to contribute to OA development and progression by primarily inducing catabolic responses in chondrocytes.^8,90–95^ Such responses converge on *Hif2α*, *Runx2*, and inflammatory mediators that lead to cartilage ECM degradation through the increased expression of MMPs and ADAMTS activity.^80,96–99^ Recent studies of genome-wide association screens (GWAS) that have been performed on large numbers of OA and control populations throughout the world have confirmed over 80 gene mutations or single-nucleotide polymorphisms (SNPs) involved in OA pathogenesis. Some of the genes are important structural and ECM-related factors (*Col2a1*, *Col9a1*, and *Col11a1*), and critical signaling molecules in the Wnt (*Sfrp3*), bone morphogenetic protein (BMP) (*Gdf5*), and TGF-β (*Smad3*) signaling pathways; most of these genes have been previously implicated in OA or articular cartilage and joint maintenance by studies using mouse models of induced genetic alteration- or surgically induced OA.^100–106^ A recent arcOGEN Consortium genome-wide screen study^107^ identified new SNPs in several genes, including GNL3, ASTN2, and CHST11. These findings need to be verified by further studies.

## Mouse models for OA research

### DMM model

DMM was developed 10 years ago and is a well established surgical OA model in mice and rats. It is widely used to study OA initiation and progression in combination with transgenic mouse models and aging and obesity models. DMM surgery was performed by transection of the medial meniscotibial ligament (MMTL).^26,27^ Briefly, following the initial incision, the joint capsule on the medial side was incised using scissors to expose either the intercondylar region or the MMTL, which anchors the medial meniscus (MM) to the tibial plateau. The MMTL was visualized under a dissection microscope and the MMTL was cut using micro-surgical scissors, releasing the ligament from the tibia plateau thus destabilizing the medial meniscus. Closure of the joint capsule and skin was with a continuous 8–0 tapered Vicryl suture. As a control for DMM studies, sham surgery was performed by only exposing the medial side of knee joint capsule. Because of the medial displacement of the meniscus tissue, greater stress occurred on the posterior femur and central tibia, especially on the medial side.^108^ Histology demonstrated the severity of OA lesions at 4-weeks post-surgery with fibrillation of the cartilage surface. Cartilage destruction and subchondral bone sclerosis developed 8 weeks post-surgery and osteophyte formation was seen 12-weeks post- surgery.^98,109–111^

### Aging model

As a degenerative disease, OA always occurs in elderly populations; thus, aging is a major risk factor for the most common form in humans, spontaneous OA. Several laboratory animals develop spontaneous OA, which approximates the stages of human OA progression. These animal models are valuable tools for studying natural OA pathogenesis.^112,113^ The most commonly used inbred strain of laboratory mouse is C57/BL6; these mice usually develop knee OA at about 17 months of age.^112^ The STR/ort mouse is one strain that easily develops spontaneous OA. It requires 12–20 weeks for STR/ort mice to develop articular cartilage destruction.^114–116^ This may be partially due to their heavier body weight compared with other mouse strains. Given the background genetic consistency, although aging OA models have many advantages, it normally requires at least one year for mice to model the disease. Therefore, surgically induced OA models^107,117^ and genetic mouse models are preferred in recent decades for their relatively fast induction for use as aging models for the study of OA lesions.

In addition to the mouse, the Dunkin Hartley guinea pig provides an aging model widely used to study OA development.^118^ The Dunkin Hartley guinea pig can develop a spontaneous, age-related OA phenotype within 3 months. The severity of OA lesions increases with age, and moderate to severe OA is observable in 18-month-old animals. Histological analysis demonstrated that the spontaneous OA progression in Dunkin Hartley guinea pig resembles that of humans. Thus, the Dunkin Hartley guinea pig is a useful animal to study the pathogenesis and evaluation of potential treatments for human OA.

### Obesity model

It has become evident that obesity contributes to a variety of musculoskeletal diseases, particularly OA, because of inflammatory and metabolic responses.^119^ Together with surgically induced injury and genetic models, mouse obesity models are widely used to explore the mechanisms of obesity-induced OA. The obese mouse model is induced by a high-fat diet, in which 60% of calories are derived from fat as opposed to the normal 13%.^120^ The entire joint tissue, but especially synovium tissue, is affected by the high-fat diet. A synovial inflammation phenotype has been independently reported by different laboratories.^54^ An elevated systemic inflammation was observed in obese mice following DMM surgery. Serum levels of pro-inflammatory factors, including interleukin-12p70,^54^ interleukin-6, TNFα and several other chemokines, were increased, suggesting a role for obesity in the development of post-traumatic OA (PTOA).

### Genetic mouse models

Genetic mouse models have recently become widely used to investigate the cellular and molecular mechanisms of OA development. Based on the GWAS studies of human patients, mutant mouse strains were generated carrying either mutant genes or SNPS. For example, *Del1*^+/-^ mice carried a mutation in the collagen II gene. Both *Del1*^*+/-*^ mice and *Col9a1*^*−/−*^ mice developed spontaneous OA.^121^ Because cartilage functions as a skeletal architect, conventional gene deletion approaches have the drawback of causing embryonic lethality or severe skeletal deformation. To overcome embryonic lethality and bypass the limits of constitutive gene knockout (KO), inducible conditional KO technology has been widely used. This usually combines Cre-loxP gene targeting with tamoxifen-induced nuclear translocation of CreER fusion protein driven by tissue-specific promoters. The *Col2a1-Cre*^*ERT2*^, *Agc1-Cre*^*ERT2*^ and *Prg4-Cre*^*ERT2*^ transgenic mice^122–124^ have become powerful tools for targeting joint tissue to study the mechanism of OA development. Based on the gene expression pattern, both *Col2a1* and *Agc1* can efficiently target chondrocytes in the growth plate cartilage, articular cartilage and temporomandibular joint. Because *Agc1* is expressed more robustly than *Col2a1* in adult cartilage tissue, *Agc1* is expected to better target chondrocytes in adult mice.^123^ In addition to chondrocytes, *Agc1* were also reported to target nucleus pulposus tissue in the intervertebral disc.^123^ *Prg4* only targets the superficial layer of articular chondrocytes.^124^ It needs to be emphasized that all of these genetic tools are used to address the importance of cartilage tissue in OA development. Additional CreER transgenic mice need to be developed to efficiently target subchondral bone, synovial tissue and meniscus.

Using these transgenic mice, specific genes have been studied in chondrocyte-specific experiments to dissect their role in OA. *In vivo* studies employing mutant mice suggest that pathways involving (i) receptor ligands, such as TGF-β1, Wnt3a, and Indian hedgehog, (ii) signaling molecules, such as Smads, β-catenin, Runx2 and HIF-2α and, (iii) peptidases, such as MMP13 and ADAMTS4/5, have some degree of involvement in OA development. Table 1 summarizes the mutant lines available for OA study.

TGF-β and its downstream molecules have important roles in OA pathogenesis. Mutations of *Smad3*, a central molecule in TGF-β signaling, have been found in patients with early-onset OA.^131–133^ It has been known for years that TGF-β promotes mesenchymal progenitor cell differentiation and matrix protein synthesis and inhibits chondrocyte hypertrophy. TGF-β signaling may play differential roles in joint tissues during OA development. For example, global deletion of *Smad3* causes chondrocyte hypertrophy and OA-like articular cartilage damage.^6^ The deletion of *Tgfbr2*, encoding for type II TGF-β receptor,^91^ or *Smad3*^12^ in articular chondrocytes also led to an OA-like phenotype. In contrast, the activation of TGF-β signaling in mesenchymal progenitor cells of subchondral bone also caused OA-like lesions.^134^ These findings suggest that TGF-β signaling may have differential roles in various joint tissues^135^ and that therapeutic interventions targeting TGF-β signaling may require a tissue-specific approach.

## Techniques for OA studies

### *In vitro* studies

#### *In vitro* articular chondrocyte isolation and culture

To investigate signaling mechanisms in articular cartilage, primary human articular chondrocytes will be obtained from surgically discarded cartilage tissues. Briefly, full-thickness sections of cartilage are excised from the subchondral bone. The cartilage pieces will be digested for about 15 h using a digestion buffer. The isolated cells will be then collected and filtered to remove undigested tissue and debris, and washed with Hanks' buffered salt solution. The cells will be then re-suspended in chondrocyte basal medium and plated in high density monolayer cultures as shown in Table 2.^136,137^ Human articular chondrocytes can also be cultured in three dimensions. Briefly, 4×10^6^ freshly isolated human articular chondrocytes will be re-suspended in alginate solution and the cell suspension is added drop-wise into 102 mmol·L^−1^ CaCl_2_ to form beads. After washing the beads with 0.15 mol·L^−1^ NaCl and basal medium, the chondrocytes encapsulated in alginate beads will be cultured in three dimensions with basal medium.^138,139^

#### *In vitro* human articular cartilage explant culture

Osteochondral tissues from radiographically and anatomically normal joints will be obtained from patients with different surgeries, such as oncologic surgical procedures, meniscal tear repair or total knee joint replacement. The collected osteochondral tissues will be first washed with sterile phosphate-buffered saline (PBS). Fresh cartilage samples will be harvested from the femoral condyle using a 6 mm diameter biopunch. The cartilage explants will be cultured in chondrocyte basal medium.^140^

### Histology/histomorphometry

Knee cartilage samples to be used for histological and histomorphometric analyses will be fixed in 10% neutral buffered formalin (NBF), decalcified in 14% EDTA for 10 days and embedded in paraffin. The paraffin-embedded samples will be cut into 5 μm sections and stained with Alcian blue/Hematoxylin-Orange G (ABH) or Safranin O/Fast green to determine changes in architectures of cartilage, bone, and synovial tissues throughout OA progression. Quantitative histomorphometric analyses of ABH-stained sections can be performed using a Visiopharm analysis system.^141^ Using this system, high resolution digital images of histology slides can be obtained. Cartilage thickness will be measured from the middle of the femoral and tibial condyles. Cartilage area will be traced from both articular cartilage surfaces. The tidemark will be used to delineate the upper and deep zone of articular cartilage.^91,93^

### OARSI score system

Several scoring systems have been developed to semi-quantify the severity of OA lesions of the knee. A scoring system recommended by the Osteoarthritis Research Society International (OARSI) society is based on continuous histological staining of the knee joint. A 0–6 subjective scoring system, as shown in Table 3, is applied to all four quadrants through multiple step sections of the joint. Sagittal sections obtained every 80 μm across the medial femoral-tibial joint will be used to determine the maximal and cumulative scores.^142^

### Nanoindentation

It is necessary to understand changes in mechanical properties of OA cartilage across multiple length scales because they directly reflect cartilage functional changes during degradation.^143^ Atomic force microscopy (AFM)-based nanoindentation is well-suited for evaluating changes at a nm-to-μm scale that is comparable to the sizes of matrix molecules and cells.^144^ For AFM-nanoindentation measurement, a microspherical or a pyramidal tip is programmed to indent the sample tissues, cells or tissue sections to a pre-set force or depth. An effective indentation modulus can be calculated by fitting the loading portion of each indentation force versus depth curve to the elastic Hertz model.^145^ The use of nanoindentation over the past decade has uncovered many new aspects of cartilage structure-mechanics relationships and OA pathomechanics. Highlights among these include micromechanical anisotropy and heterogeneity of healthy and OA cartilage^146^ or meniscus,^147^ cartilage weakening in spontaneous^148,149^ and post-traumatic^150–152^ OA, mechanics of individual chondrocytes,^151,153^ and quality evaluation of engineered neo-tissues.^154–156^

Notably, AFM-nanoindentation has made it possible to study the mechanical properties of murine cartilage. Previously, the ~100 μm thickness of murine cartilage prevented such attempts. Because *in vivo* OA studies are largely dependent on murine models,^157^ nanoindentation provides a critical bridge across two crucial fields of OA research: biology and biomechanics. The benefit of nanoindentation for murine model studies has been demonstrated by a number of recent studies. For example, cartilage in mice lacking collagen IX (*Col9a1*^*−/−*^)^148^ showed abnormally higher moduli, while those lacking lubricin (*Prg4*^*−/−*^)^158^ or chondroadherin (*Chad*^*−/−*^)^159^ showed lower moduli. *Col9a1*^*−/−*^ and *Prg4*^*−/−*^ mice also developed macroscopic signs of OA,^148,158^ underscoring the high correlation between abnormalities in cartilage biomechanics and OA. Li *et al.* also recently demonstrated the applicability of nanoindentation to the murine meniscus.^160^ Further applications of nanoindentation to clinically relevant OA models, such as the DMM model,^110^ hold the potential of assessing OA as an entire joint disease through biomechanical symptoms in multiple murine synovial tissues.

Two other recent technological advances provide paths to further in-depth studies. First, Wilusz *et al.*^161^ stained cartilage cryosections with immunofluorescence antibodies of the pericellular matrix signature molecules, type VI collagen and perlecan.^162^ Using immunofluorescence guidance, nanoindentation was used to delineate the mechanical behavior of cartilage pericellular matrix and ECM,^161–163^ and to reveal the role of type VI collagen in each matrix by employing *Col6*^*−/−*^ mice.^164^ Therefore, it is now possible to directly examine the relationships across micro-domains between biochemical content and biomechanical properties of cartilage,^161^ meniscus^165^ or other synovial tissues *in situ*. Second, Nia *et al.*^166^ converted the AFM to a high-bandwidth nanorheometer. This tool enabled separation of the fluid flow-driven poroelasticity and macromolecular frictional intrinsic viscoelasticity that govern cartilage energy-dissipative mechanics.^166–168^ Hydraulic permeability, the property that regulates poroelasticity, was found to be mainly determined by aggrecan rather than collagen^169^ and to change more drastically than modulus upon depletion of aggrecan.^166,170^ This new tool provides a comprehensive approach beyond the scope of elastic modulus for assessing cartilage functional changes in OA.

## Molecules mediating OA pain

The perception of OA pain is a complex and dynamic process involving structural and biochemical alterations at the joint as well as in the peripheral and central nervous systems. While there have been extensive studies of mediators of OA joint degeneration, only recently have studies begun to characterize biochemical influences on and in the peripheral and central nervous systems in OA. In this regard, OA appears to show similarities and differences with other conditions causing pain.^171,172^ There are a wide variety of signaling pathways linked to joint destruction and/or pain. In this section we will discuss three emerging and highly relevant pathways that provide insight into the mechanisms underlying OA pain.

### Chemotactic cytokine ligand 2/chemokine (C–C motif) receptor 2

Chemotactic cytokine ligand 2 (CCL2), also known as monocyte chemoattractant protein 1 (MCP-1), is well-known to mediate the migration and infiltration of monocytes and macrophages by signaling through chemokine (C–C motif) receptor 2 (CCR2).^173^ In arthritis, CCL2 promotes inflammation of the joint.^174^ Evidence also suggests that CCL2 is an important mediator of neuroinflammation.^175,176^ In neuropathic pain, CCL2 expression is increased in microglia and in sensory neurons in the dorsal root ganglia (DRGs), where CCL2 can be further transported and released into central spinal nerve terminals. Increased CCL2/CCR2 signaling has been correlated with direct excitability of nociceptive neurons and microglial activation, leading to persistent hyperalgesia and allodynia.^177,178^

In a DMM mouse OA model, CCL2 and CCR2 levels were elevated in DRGs at 8 weeks post surgery, correlating with increased OA-associated pain behaviors. Increased CCL2 and CCR2 levels in the DRG were thought to mediate pro-nociceptive effects both by increasing sensory neuron excitability through CCL2/CCR2 signaling directly in DRG sensory neurons and through CCL2/CCR2-mediated recruitment of macrophages in the DRG. Compared with wild-type mice, *Ccr2*-null mice showed reduced pain behaviors following DMM with similar levels of joint damage.^179^ Although CCR2 antagonists are currently being assessed in clinical studies, no clinical studies have targeted CCL2 or CCR2 in OA pain.^180^

### Nerve growth factor/tropomyosin receptor kinase A

In both clinical and animal studies, the targeted inhibition of nerve growth factor (NGF) and inhibition of its cognate receptor, tropomyosin receptor kinase A (TrkA), reduced OA pain. Clinically, the systemic administration of NGF caused persistent whole-body muscle hyperalgesia in healthy human subjects,^174,177^ while anti-NGF antibody, tanezumab, therapy significantly reduced OA pain.^181–184^ There are a number of potential mechanisms through which NGF mediates pain. Over-expressed NGF in peripheral tissues can bind directly to TrkA at sensory neuron nerve terminals and be retrogradely transported to the DRG. There it stimulates sensory neurons to activate mitogen-activated protein kinase (MAPK)/extracellular signal-regulated kinase (ERK) signaling.^185^ The activation of the NGF-MAPK/ERK axis upregulates the expression of pain-related molecules, including transient receptor potential cation channel subfamily V member I (TRPV1), substance P, calcitonin gene-related peptide (CGRP), brain-derived neurotrophic factor (BDNF), and nociceptor-specific ion channels, such as Ca_v_ 3.2, 3.3, and Na_v_1.8.^186–188^

In addition to direct signaling of sensory neurons, NGF promotes algesic effects by targeting other cell types. For example, NGF/TrkA signaling occurs in mast cells, triggering release of pro-inflammatory and pain mediators, including histamine and prostaglandins, in addition to NGF.^186,189^ NGF signaling is upregulated by pro-inflammatory mediators, and NGF promotes leukocyte chemotaxis and vascular permeability, further stimulating inflammation.^190–192^ NGF/TrkA signaling further promotes angiogenesis and nerve growth. The process of angiogenesis is not only inflammatory, but also serves as a track for nerve growth into the joint.^193^

Given the high efficacy of targeting NGF in a clinical study on reducing OA pain, it is of great interest to further define NGF/TrkA pain signaling mechanisms and to find additional therapeutic targets in this pathway. Recent evidence indicates that loss of PKCδ signaling significantly increases both NGF and TrkA in the DRG and synovium, is associated with increased MAPK/ERK signaling at the innervating DRGs, and is associated with OA hyperalgesia.^194^ However, in recent clinical studies, a small population of patients treated with systemic anti-NGF therapy exhibited rapid progression of OA and were more prone to bone fractures.^195^ Considering the analgesic effects by anti-NGF therapy on OA-associated pain, understanding of the precise roles of the NGF/TrkA pathway in different joint tissues in OA and OA-associated pain is of great interest.

### ADAMTS5

The use of *Adamts5* KO mice and therapeutic treatment with anti-ADAMTS5 antibody in wild-type mice produce inhibition of ADAMTS5 signaling/expression in the DMM model, resulting in reduction of both joint degeneration and pain.^98,196,197^ ADAMTS5 is a major aggrecanase, and because aggrecan is a major component of the proteoglycans in cartilage that provides compressive resistance, ADAMTS5 is thought to be a critical mediator of cartilage degeneration during the development of OA.^198^ Although variations in pain signaling can be independent from the degree of joint degeneration, the use of *Adamts5* KO mice and direct inhibition of joint degeneration with anti-ADAMTS5 antibody may provide insight into how joint degeneration produces OA pain. For example, hyalectan fragments generated by ADAMTS5 have been suggested to directly stimulate nociceptive neurons as well as glial activation, promoting increased pain perception.^196,199^ Furthermore, inhibition of ADAMTS5 following DMM resulted in reduced levels of CCL2 in DRG neurons, thus suggesting a role for CCL2 in OA-specific pain.^197^

### Pain-related behavior tests

Pain is the most common reason patients seek medical treatment and is a major indication for joint replacement surgery.^200,201^ Therefore, evaluating pain in pre-clinical animal models is of critical importance to better understand mechanisms of and to develop treatments for OA pain. The evaluation of OA pain in animals involves indirect and direct measures.

Recognizing pain as a clinical sign and quantitatively assessing pain intensity are essential in research for effective OA pain management. Rodent animal models are routinely used for basic and pre-clinical studies because of the relatively low cost of animal maintenance, the abundance of historical data for comparison, and smaller amounts of drugs required for experimental studies. For pain measurements, rodents have advantages over other small animal models, such as rabbits, which present challenges to obtain a pain response and are immobile if startled by an unfamiliar observer. Mice are usually used for the development of genetically engineered strains to enable molecular understanding of OA progression and pain *in vivo*.^202^ Larger animals, including dogs, sheep, goats, and horses are also sometimes used for modeling OA pain.^202,203^

A wide range of direct and indirect measures of pain are used in small animal models of OA. Indirect and/or direct measures of pain include static or dynamic weight bearing, foot posture, gait analysis, spontaneous activity, as well as sensitivity to mechanical allodynia, mechanical hyperalgesia, and thermal, and cold stimuli.^202,203^ Among indirect tests involving pain-evoked behaviors, mechanical stimuli may be the most correlated with OA pain. A commonly used measure of indirect pain is the von Frey test for mechanical allodynia using filaments to assess referred pain.^186,194,196,202,204^ Direct mechanical hyperalgesia is performed using an analgesymeter for paw pressure pain threshold. Additional direct measures of OA pain include the hind limb withdrawal test, vocalization evoked by knee compression on the affected knee, the struggle reaction to knee extension, and ambulation and rearing spontaneous movements.^194,202,203^ Weight-bearing and gait analyses may have important translational relevance for assessing OA pain because these tests are also used to assess clinical OA pain.^203^ However, obtaining clear pain responses from weight bearing or gait is challenging when using the unilateral DMM mouse model because the nature of OA pain is a dull pain unlike that of, for example, sharp inflammatory pain.

In large animals, pain behavior testing is more challenging and there is no consensus for the best method of evaluating pain.^202^ However, dogs, the most commonly used large animal, have been suggested to provide the best predictive modeling for OA pain translated into the clinical setting.^205^ Methods used for assessing pain in large animals are restricted to assessing degree of lameness, gait analysis, and subjective rating scales, which assess descriptors of pain similar to those of humans.

Overall, there is a wide range of pain-behavior tests for small and large animal models. Although no animal model or pain behavior test perfectly translates to OA-associated pain in patients, these tests yield a valuable understanding of the mechanisms of OA pain and allow assessment of treatments for relief from OA-associated pain. Rodents will continue to be widely used for basic OA pain research, but large animals continue to be important because of their greater potential for modeling clinical OA pain.

## Future perspective

Although significant progress has been made in OA research in recent years, very little is yet known about the molecular mechanisms of OA initiation and progression. OA is a heterogeneous disease caused by multiple factors. One important potential factor for OA development is Runx2, which is upregulated in several OA mouse models and in cartilage samples derived from patients with OA disease.^7,8,11,13,91^ Key questions that need to be addressed are: (1) Is Runx2 a central molecule mediating OA development in joint tissue?; and (2) Could manipulation of Runx2 expression be used to treat OA disease? OA is a disease affecting the entire joint, including articular cartilage, subchondral bone, synovial tissues and menisci. In which of these joint tissues OA damage first occurs during disease initiation is currently unknown; this is important because it is directly related to OA treatment. In addition, the interplaying mechanisms among different OA symptoms, such as articular cartilage degradation, osteophyte formation, subchondral sclerosis and synovial hyperplasia, await clarification. The understanding of the molecular mechanisms underlying these issues will accelerate the development of novel therapeutic strategies for OA.

## Figures and Tables

**Table 1:** Available transgenic mouse models for osteoarthritis research

Gene	Targeting tissue	Pathway
*Del1*^125^	Global	ECM
*Col9a1*^126^	Global	ECM
*Tgfbr2*^90^	4xMRE	TGF-β
*Smad3*^6^	Global	TGF-β
*Smurf2*^127^	Col2-Cre	TGF-β
*Tgfbr2*^91^	Col2-CreER	TGF-β
*Frzb*^128^	EIIaCre	Wnt
*β-catenin*^7^	Col2-CreER	Wnt
*Rbpjk*^93^	Prx1Cre	Notch
*Fgfr1*^129^	Col2-CreER	FGF
*Smo*^8^	Col2-Cre	Ihh
*Runx2*^11^	Global	
*Hif2a*^9^	Global	
*Mmp13*^97^	Global	
*Mmp13*^130^	Col2-CreER	
*Adamts5*^98^	Global	

Abbreviations: ECM, extracellular matrix; FGF, fibroblast growth factor; Ihh, Indian Hedgehog; TGF-β, transforming growth factor-β.

**Table 2:** Monolayer culture conditions for human primary articular chondrocytes

Plate type	Volume per well	No. of cells per well
6-well	2.5 mL	1×10^6^
12-well	1 mL	4×10^5^
24-well	0.5 mL	2×10^5^

**Table 3:** The recommended semi-quantitative scoring system^143^

Grade	Osteoarthritic damage
0	Normal
0.5	Loss of Safranin O without structural changes
1	Small fibrillations without loss of cartilage
2	Vertical clefts down to the layer immediately below the superficial layer and some loss of surface lamina
3	Vertical clefts/erosion to the calcified cartilage extending to <25% of the articular surface
4	Vertical clefts/erosion to the calcified cartilage extending to 25%–50% of the articular surface
5	Vertical clefts/erosion to the calcified cartilage extending to 50%–75% of the articular surface
6	Vertical clefts/erosion to the calcified cartilage extending to >75% of the articular surface
